# Neurocognitive Complications after Ventricular Neuroendoscopy: A Systematic Review

**DOI:** 10.1155/2020/2536319

**Published:** 2020-03-25

**Authors:** Jehuda Soleman, Raphael Guzman

**Affiliations:** ^1^Department of Neurosurgery, University Hospital of Basel, Basel, Switzerland; ^2^Division of Pediatric Neurosurgery, Children's University of Basel, Basel, Switzerland; ^3^Faculty of Medicine, University of Basel, Basel, Switzerland

## Abstract

In recent years, neuroendoscopic treatment of hydrocephalus and various ventricular pathologies has become increasingly popular. It is considered by many as the first-choice treatment for the majority of these cases. However, neurocognitive complications following ventricular neuroendoscopic procedures may occur leading mostly to amnesia, which might have a grave effect on the patient's quality of life. Studies assessing neurocognitive complications after ventricular neuroendoscopic procedures are sparse. Therefore, we conducted a systematic review assessing the available literature of neurocognitive complications and outcome after ventricular neuroendoscopy. Of 1216 articles screened, 46 were included in this systematic review. Transient and permanent neurocognitive complications in 2804 ventricular neuroendoscopic procedures occurred in 2.0% (*n* = 55) and 1.04% (*n* = 28) of the patients, respectively. Most complications described are memory impairment, followed by psychiatric symptoms (psychosyndrome), cognitive impairment not further specified, declined executive function, and confusion. However, only in 20% of the series describing neurocognitive complications or outcome (*n* = 40) was neurocognition assessed by a trained neuropsychologist in a systematic manner. While in most of these series only a part of the included patients underwent neuropsychological testing, neurocognitive assessment was seldom done pre- and postoperatively, long-term follow up was rare, and patient's cohorts were small. A paucity of studies analyzing neurocognitive complications and outcome, through systematic neuropsychological testing, and the correlation with intraoperative lesions of neuronal structures (e.g., fornix) exists in the literature. Therefore, the neurocognitive and emotional morbidity after ventricular neuroendoscopic procedures might be underestimated and warrants further research.

## 1. Introduction

Ventricular neuroendoscopy, for the treatment of occlusive, and also nonocclusive, hydrocephalus, colloid cysts (CC), intraventricular cysts, fourth ventricle outlet obstruction (FVOO), and intraventricular tumors has become increasingly popular over the last two decades [1–4]. Various ventricular endoscopic procedures, such as third ventriculostomy (ETV), CC resection or aspiration, tumor biopsy or resection, septum pellucidotomy, and foraminoplasty or stenting, have been described. Endoscopic procedures are often described as minimally invasive, since they lead to lower morbidity and mortality rates when compared to open microsurgical procedures [5, 6]. In addition, endoscopic treatment of hydrocephalus is considered preferable to the placement of ventriculoperitoneal shunt (VPS) in patients above the age of six months, since it is at least as efficient and it avoids a lifetime shunt dependency and associated complications, occurring sometimes years after VPS placement [1, 7]. Despite the growing preference of neuroendoscopic procedures for the treatment of hydrocephalus and intraventricular lesions, only few studies analyze variables such as cognitive and emotional deficits following these procedures [3, 4, 8–16]. In addition, the very few studies assessing for neurocognition in a systematic manner do not focus on neurocognitive decline caused by the surgery itself, but rather on improvement in neurocognitive outcome. Neurocognitive complications after ventricular neuroendoscopy are difficult to assess, since hydrocephalus and the lesions within the ventricles might be the reason for the neurocognitive impairment. Nevertheless, it seems that neurocognitive complications, due to intraoperative damage to the fornix, mamillary bodies, anterior thalamus, hypothalamus, and hippocampal formation and fibers, are underestimated and seldom assessed through systematic neuropsychological test batteries [2, 15, 17]. We provide a systematic review summarizing the rate of cognitive complications after ventricular neuroendoscopic procedures. First, the anatomical background of ventricular structures involved in neurocognition is described. Thereafter, ventricular pathologies potentially causing neurocognitive decline are discussed. Following, the results of studies evaluating neurocognition based on systematic neurocognitive test batteries, concluded by trained neurophysiologist, are discussed in more detail. Finally, ways to avoid neurocognitive complications during ventricular neuroendoscopy and suggestions for future research are presented and discussed.

## 2. Methods

**Figure 1:**
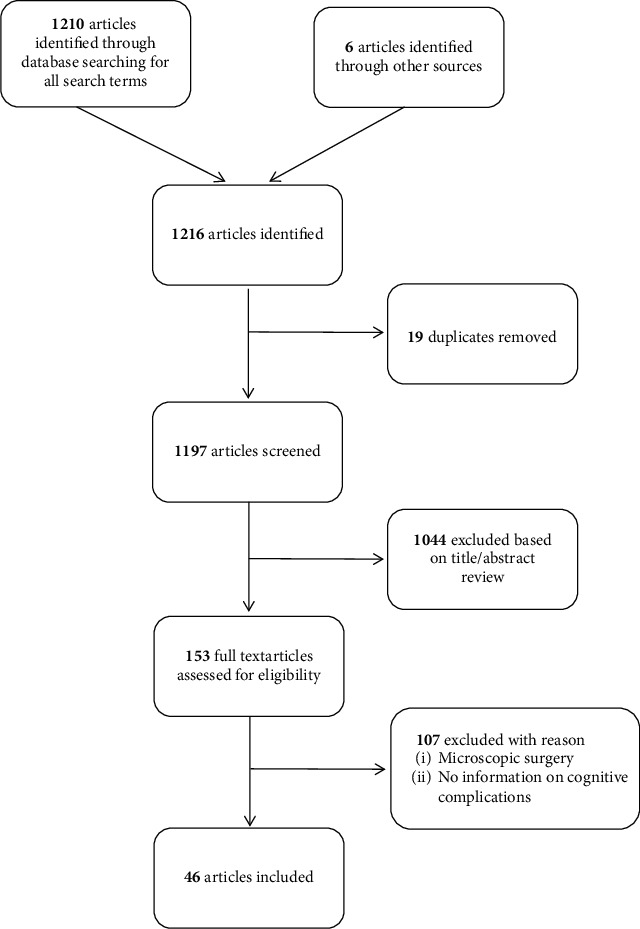
Selection of articles included in this review.

References for this review were identified by searching of PubMed between 1960 and 2019. Terms inserted were “neuroendoscopy AND complications”, “neuroendoscopy AND cognitive outcome”, “neuroendoscopy AND memory”, “neuroendoscopy AND quality of life”, “neuroendoscopy AND cognition”, “neuroendoscopy AND neuropsychological outcome”, “endoscopic third ventriculostomy AND neuropsychology”, “endoscopic third ventriculostomy AND neurocognition”, “endoscopic third ventriculostomy AND neurocognitive”, “colloid cyst AND neuropsychology”, “colloid cyst AND neurocognition”, and “colloid cyst AND neurocognitive” with restrictions to English language, case reports, clinical trials, controlled clinical trials, meta-analyses, randomized controlled trials, reviews, and systematic reviews. Abstracts were reviewed by the authors, duplicates were removed, and the final list of references was generated (Figure 1). We included only studies, where cognitive complications, cognitive outcome, or lesions to neurocognitive anatomical structures (e.g., fornix and mamillary bodies), after ventricular neuroendoscopy for various indications were described. Inclusion was not limited to a specific age group; therefore, studies of all age spans (adults, pediatric, or both) were included. The review was performed in accordance with the Preferred Reporting Items for Systematic Reviews and Meta-Analyses (PRISMA) guidelines.

## 3. Results

After searching for all terms, 1210 records were identified by the database and 6 additional records were identified through references within selected records. After removal of 19 duplicates, 1197 records were screened. Based on title or abstract review, 1044 records were excluded. Out of the remaining 153 records, 107 were excluded with reason resulting in 46 articles (Figure 1).

Out of over 150 screened series, discussing complications after ventricular neuroendoscopy, only 40 specifically describe postoperative cognitive complications [2–5, 10, 12, 14–47], of which only eight (20%) evaluate postoperative neurocognitive outcome in a systematic manner. In most of these eight series, not all of patients underwent neuropsychological testing, neurocognitive assessment was seldom done pre- and postoperatively, long-term follow up was rare, and patient's cohorts were small. Three case reports [8, 9, 11] and three reviews [1, 48, 49] describing or discussing postoperative cognitive complications were included in this systematic review as well. The vast majority of the included series were of retrospective manner, while 28 (70%) of the included studies describe the outcome in less than 50 patients, five (12.5) include 50-100 patients, four (10%) 100-200 patients, two (5%) 200-500 patients, and one (2.5%) more than 500 patients (Table 1). In 25 studies, a rigid endoscope was used; in four studies, a flexible endoscope was used; and in six studies, both flexible and rigid endoscopes were used, while in 6 studies, the type of endoscope used was not described (Table 1).

Table 1 presents the 40 included series describing neurocognitive complications, of which 8 assess for neurocognitive outcome through specific neuropsychological test batteries [3, 4, 10, 12–16]. Transient and permanent neurocognitive complications in 2804 ventricular neuroendoscopic procedures occurred in 2.0% (*n* = 55) and 1.04% (*n* = 28) of the patients, respectively. Most complications described are memory impairment, followed by psychiatric symptoms (psychosyndrome), cognitive impairment not further specified, declined executive function, and confusion (Table 1). Neurocognitive complication rates for specific types of ventricular neuroendoscopic procedures are presented in Table 2.

## 4. Discussion

### 4.1. Structures Involved in Neurocognition at Risk during Ventricular Neuroendoscopy

Based on the very limited and low-quality literature available, it seems that the most frequent neurocognitive complication after ventricular neuroendoscopy is memory impairment, specifically anterograde amnesia, while decline in executive function and psychiatric disorders are described as well [1, 6, 8, 9, 11, 13, 16, 20, 21, 23, 29, 32, 34, 40, 46, 50]. To note, many patients with ventricular pathologies present with memory impairment to begin with; therefore, the assessment of postoperative memory impairment is often hindered, especially when neuropsychological assessment, by a specialized neuropsychologist, before and after surgery is not performed [1, 15, 51]. This might also explain the fact that some authors feel that neurocognitive complications due to surgery are often neglected or not realized and are therefore underestimated [1, 17, 52]. In addition, lesions of important ventricular structures caused by surgical procedures are rarely assessed for and seldom described within reports in the literature, although such lesions potentially lead to incriminating neurocognitive morbidity. For these reasons, the knowledge of ventricular anatomy and its adjacent neuronal structures, which are involved in important neurocognitive functions, such as memory and executive functions, is imperative. Improved knowledge of the anatomy and function of neuronal structures within the ventricle, specifically the 3^rd^ ventricle, will most probably lead to improved assessment of neurocognitive complications and their reporting in the literature after ventricular neuroendoscopy. Herein, we provide a short overview of the main structures within or in proximity to the 3^rd^ ventricle, involved in neurocognitive functions.

The roof of the third ventricle consists of the hippocampal commissure, as well as the crus and body of the fornix [53]. Within the floor of the third ventricle, the mamillary bodies are seen, while the columns of the fornix and the foramen of Monro limit the anterior wall [53]. The thalamus, hypothalamus, and further the columns of the fornix are found within the lateral wall of the third ventricle [53]. It is important to acknowledge that the fornix runs along the cranial part of the septum pellucidum. The fornix is the major tract connecting the hippocampal formation to the mamillary bodies, the diencephalon (consisting amongst others of the hypothalamus and thalamus), and the medial temporal regions [54–57]. All of these structures are believed to be involved in memory and other important cognitive functions such executive functions. Lesions to these structures are often associated with temporal lobe and diencephalic amnesia beyond executive function disorder [54, 55, 57, 58]. Some fibers of the limbic system (fornix-hippocampus-mamillary bodies) seem to be linked and connected with the amygdaloid complex and the orbitofrontal cortex both discussed in control of emotions, decision-making, and social cognition [57]. Thus, emotional disturbances, mood changes, and psychiatric symptoms might occur due to lesions to the fornix, hippocampal formation, anterior thalamus, hypothalamus, or mamillary bodies [57]. However, such symptoms could also be due to psychological factors such as psychogenic causation of cognitive symptoms (e.g., amnesia) or stress associated with the operation itself, leading to an outburst of neuropsychiatric symptoms [57]. Damage to the forniceal pathways as the cause for retrograde amnesia was always a matter of debate. Some studies show that temporal lobe or diencephalic lesions have a stronger association with anterograde amnesia than damage to the fornix. On the other hand, some recent publications showed convincing data that damage to the forniceal tracts causes memory impairment [55, 58, 59]. In addition, atrophy of the mamillary bodies, usually occurring due to fornix lesions, was found to be strongly associated with memory impairment [55, 59]. A correlation between fornix damage on postoperative MRI after colloid cyst (CC) resection and memory impairment was seen as well, underlining the evidence that damage to the fornix does lead to memory impairment [55, 58]. Whether unilateral or only bilateral damage to the fornix leads to memory and/or cognitive impairment remains ambiguous [20, 51]. McMacking et al. showed that bilateral fornix damage leads to amnesia, while unilateral damage leads to selective impairment according to the side of the lesion. Some reports indicate that unilateral damage to the left fornix is sufficient to induce persistent loss of verbal memory [55]. Aggleton et al. conclude that when reviewing all CC resection cases with and without fornix damage published, it becomes difficult not to conclude that fornix damage is sufficient to induce persistent and marked loss of memory [55]. Further, based on the provided literature, it seems that damage to the mamillary bodies, anterior thalamus, hypothalamus, and hippocampal formation can lead to memory and cognitive impairment as well [54–56, 59]. Lastly, little is known about the role of the median eminence, a circumventricular organ located in the premammillary region and visible only under fluorescein-guided endoscopy [60]. This structure is “regularly” destroyed during endoscopic third ventriculostomy, and the cognitive ramifications of its destruction remain unknown.

### 4.2. Ventricular Pathologies Leading to Neurocognitive Impairment

Various ventricular pathologies are known to cause neurocognitive impairment through compression of intra- or paraventricular structures (e.g., fornices, mamillary bodies, hypothalamus, and thalamus), increased intracranial pressure, or impairment of blood flow leading to atrophy of intra- or paraventricular structures (e.g., fornices, mamillary bodies, hypothalamus, and thalamus).

Hydrocephalus is known to cause neurocognitive impairment, especially of anterograde memory in combination with frontal executive function [12, 51, 61, 62]. This is most probably due to increased intracranial pressure, leading to direct pressure on important structures such as the fornix, hypothalamus, mamillary bodies, hippocampus, corpus callosum, and other connecting white matter tracts.

Colloid cysts (CC) are benign cysts typically arising from the roof of the third ventricle in great proximity to the fornices. Therefore, even small cysts can cause neurocognitive impairment due to local compression of the fornix. Large cysts often cause occlusive hydrocephalus leading to cognitive impairment in combination with local fornix compression [4, 15, 51, 55, 56].

Ventricular tumors causing obstructive hydrocephalus, local compression of important structures, especially those involving the 3^rd^ ventricular floor or wall, or even causing blood flow impairment or intraventricular or intraparenchymal hemorrhage typically cause amongst others neurocognitive symptoms [51, 57].

Similarly, intraventricular arachnoid or choroid plexus cysts typically cause cognitive impairment, due to either hydrocephalus and increased intracranial pressure or local compression of important intra- and paraventricular structures.

Because most ventricular pathologies lead to neurocognitive impairment, the assessment of neurocognitive outcome and complication rate after neuroendoscopic treatment of these patients is difficult. It is therefore imperative that patients with ventricular pathologies undergo neuropsychological evaluation, through a validated neuropsychological test battery, by trained neuropsychologists, before and after neuroendoscopic surgery (Table 3). In addition, it would be of great value if these neuropsychological test batteries would be unified within the different research groups so that better understanding and comparison between the neurocognitive results would be possible. Studies assessing for the correlation between intraoperative fornix injuries (and other structures such as the hypothalamus, mamillary bodies, and vascular structures); postoperative magnetic resonance imaging (MRI) including MR angiography, diffusion weighted imaging, and diffusion tensor imaging (DTI) [63]; and neurocognitive outcome would be highly relevant [17, 51, 52].

### 4.3. Neurocognitive Complications and Outcome after Ventricular Neuroendoscopy

The first series analyzing the neurocognitive outcome, through neuropsychological test batteries, was published in 2003 by Burtscher and colleagues [12]. Neuropsychological testing was done prospectively one week before ETV for late onset idiopathic aqueduct stenosis (LIAS) and on two follow-up examinations (mean after 7.5 and 81.2 weeks). Six adults with LIAS were assessed. All patients showed preoperative cognitive impairment, some of them ranging into the lowest centile scores. Impairment of anterograde memory in combination with frontal executive cognitive deficits was the most common problem. Three patients did not notice any cognitive deterioration in their daily life, even though neuropsychological testing showed clear deficits. Follow-up examinations showed good recovery of memory and other impairments in five patients and moderate recovery in one. No neurocognitive complications occurred in their series. They conclude that ETV is an effective and safe treatment for patients with LIAS, since it improves apart from somatic symptoms also neurocognition [12]. In 2008, Lacy et al. presented data on 10 adult patients undergoing ETV and neuropsychological testing [14]. They showed that 40% of the patients displayed memory and/or executive dysfunction two years after surgery, despite relatively normal ventricular size in all patients. In addition, no new insults such as stroke or brain contusion were noted on postoperative imaging. Because, preoperative neuropsychological assessment was not available, it is difficult to conclude whether these deficits were new and therefore due to surgical injuries or a persisting state due to the underlying pathology and/or the hydrocephalus. Another interesting finding was that 50% of the cohort endorsed items suggestive of depression, and 30% endorse anxiety-related symptoms. They conclude that the reason for the neurocognitive deficits is most likely multifactorial and that patients undergoing ETV should be tested for neurocognition and also for depression and anxiety [14]. Sribnick et al. in 2013 were the first group assessing neurocognitive complications in 52 patients (age 16-77 years) after endoscopic CC resection. They did not conduct neuropsychological testing in a systematic manner; however, retrospective telephone interviews were undertaken, where the patients were asked about improvement of symptoms after surgery, new symptoms, and specifically new memory problems, after surgery, the ability to return to the same job after surgery, and patients' satisfaction. They describe transient and permanent memory impairment in six (11%) patients each, while four of the patients with permanent memory impairment returned to their old job. Overall, 100% of the patients were satisfied with the operation, while 92% were able to return to work after surgery [16]. In 2014, Hader and colleagues analyzed cognitive complications and outcome after ETV in a mixed (adult and pediatric) group of 19 patients [13]. In their series, 85% of the patients showed improvement in at least one cognitive domain (intelligence, attention and concentration, verbal and visual memory, language, and executive function) after ETV. Subjectively, 69% of the patients reported improvement in cognitive function, while the rest cited no change. To note, two pediatric patients (17%) showed worsening in executive function, which potentially may be due to disruption of frontal white matter tracts due to the endoscopic approach. However, since most patients showed improvement or no change in cognition after ETV, the authors conclude that cognitive decline after ETV is uncommon in pediatric and adult patients. Additionally, they state that patients presenting with chronic obstructive hydrocephalus and history of progressive cognitive dysfunction alone may profit from ETV [13]. Hugelshofer et al. assessed 11 right-handed patients with space-occupying intraventricular cysts on their dominant side, who underwent endoscopic fenestration through a contralateral (nondominant) approach [3]. Preoperative neuropsychological assessment in 10 patients revealed cognitive impairment in eight patients, while all eight patients showed postoperative cognitive improvement after neuropsychological testing. One patient suffered transient postoperative memory deficit, which completely resolved after five days. No permanent cognitive complications were seen. They conclude that a nondominant approach for dominant-hemispheric ventricular cysts is associated with very low approach-related morbidity [3]. Ten out of 22 patients undergoing CC resection underwent neuropsychological testing in a series published in 2016 by Birski et al. [10]. In all patients, cognitive function in particular memory improved or remained unchanged after surgery. One patient suffered short-term memory impairment after surgery, which resolved within 48 hours. They conclude that endoscopic CC resection shows favorable cognitive outcome [10]. Recently, Roth et al. published their results on the cognitive outcome after resection of CC [15]. Of the 23 patients undergoing surgery for CC included, 18 underwent endoscopic surgery. Two patients experienced forniceal abrasion without any permanent cognitive impairment, while transient cognitive deficits are not described. Neurocognitive outcome (in 14 out of the 23 operated patients) was done systematically by a neuropsychologist; however, they did not distinguish endoscopically and microsurgically operated patients when presenting the data. Therefore, drawing firm conclusions for neurocognitive outcome after endoscopic resection of CC is difficult. Nevertheless, most of the patients included were treated endoscopically, and an immediate postoperative improvement in neurocognition, especially in visual memory, was seen in the majority of the operated patients. The authors conclude that surgical removal of CC leads to immediate cognitive improvement, which stabilizes over months, while further research with routine and systematic pre- and postoperative neuropsychological testing, in this group of patients, is encouraged [15]. Lastly, a study published by Vorbau et al. recently presented long-term follow-up data (15.7 years on average) of 20 patients (pediatric and adult) undergoing CC resection [4]. Five superficial fornix contusions after endoscopic removal were seen, while in one patient, severe fornix atrophy caused by chronic hydrocephalus was seen. Three patients presented with a transient psychotic syndrome, while none of the cognitive complications were permanent. Neuropsychological testing in 14 patients showed that 10 patients achieved average test results, while four patients scored borderline to abnormal test results. Since preoperative neuropsychological testing was not conducted in their study and due to the rather small patient group, they could not determine whether the poor cognitive results were due to the underlying pathology (CC, hydrocephalus) or the surgical procedure.

Benabarre et al., in 2001, published for the first time a report of a neurocognitive complication resulting from a ventricular endoscopic procedure [9]. The patient underwent an ETV for the treatment of slit ventricle syndrome, developing a severe organic personality disorder, characterized by impulsiveness, physical heteroaggressiveness, binge eating, hypersomnia, and impairment of memory and frontal executive functions. The patient showed symptoms referring to frontal lobe lesions and damage to the fornix and its connection to the hippocampus and mamillary bodies, which was confirmed by postoperative MRI. Thereafter, an additional report of a woman undergoing ETV for an AS showing severe psychotic depression, occurring gradually within three weeks after surgery, was published in 2002 by van Aalst and colleagues [8]. Finally, in 2004, a report by Bonanni et al., describing a case of permanent episodic memory impairment, associated with bulimia, after ETV, was published [11]. These case reports were of great impact, since they made neurosurgeons aware of such complications following ETV, which was and still is considered a minimal invasive and benign procedure. Very few reviews dealing with ventricular endoscopic complications discuss neurocognitive complications. Yadav et al. published two reviews on complication avoidance in endoscopic neurosurgery and specifically in ETV [48, 49]. According to Yadav et al., fornix injury is one of the most common complications of ETV and ventricular endoscopy [48]. Bouras and Sgouros published in 2011 a review on complications after ETV. Out of approximately 2800 patients in 17 studies on ETV reviewed, intraoperative neuronal injuries were reported in 0.24%. Forniceal lesions were reported in 0.04%, while out of 2.38% permanent morbidity calculated, permanent memory disorder was seen in 0.17%. The authors discuss that the reported rate of intraoperative neuronal injuries is probably underestimated [1]. Our results confirm this assumption, while based on our systematic review, most probably, postoperative neurocognitive complications are underestimated as well. Neurocognitive complications are seldom described in the framework of endoscopic outcome studies, let alone analyzed routinely and systematically by a neuropsychologist with a validated neuropsychological test battery before and after ventricular endoscopic procedures. Table 3 describes the neuropsychological test batteries, which are preformed at our institution for patients undergoing neuroendoscopy. Clearly, acknowledging the difference between disease-related and surgery-related complications remains a challenge. However, through comparison of the pre- and postoperative neuropsychological testing results, differentiating between disease- and surgery-related neurocognitive deficits is possible. Postoperative unchanged or even improved neurocognitive functions suggest that the deficits are disease-related, while new or progressing postoperative neurocognitive deficits are most probably surgery-related. Further studies, with larger patient groups, assessing neurocognition in an objective and also subjective (from the patients' point of view) manner, and with long follow-up time, are needed for us to better understand the true neurocognitive complication rate after ventricular neuroendoscopy.

### 4.4. How to Avoid Injuries of Neuronal Structures during Ventricular Neuroendoscopy

Preservation of the fornix, mamillary bodies, and all other associated “limbic” structures within or adjacent to the third ventricle during neuroendoscopic procedures is critical. Although some authors report lesions to these structures in up to 16.4% of neuroendoscopic procedures, they often remain clinically silent [39]. Based on a published meta-analysis comparing open vs. endoscopic CC resection, permanent neurocognitive morbidity after endoscopic resection occurred in 4.9% of the cases (compared to 26% of the cases in open microscopic surgery). The data of our current systematic review shows a rate of 2% transient and 1% permanent cognitive impairment after various ventricular neuroendoscopic surgeries. The reason that most intraoperative damages to neuronal structures remain clinically silent might be due to various reasons. First, minor contusion of these structures might be well tolerated by the patients remaining clinically silent. Second, some of these lesions might be only due to tension to these structures without disruption or destruction of the fibers or neurons, and therefore, clinical symptoms do not occur. Last, since in most studies systematic pre- and postoperative neuropsychological testing was not conducted, new subtle neurocognitive changes after surgery might have been missed.

The following points minimize the risk of fornix injury and injury to other neuronal structures during endoscopic procedures: The type of endoscope, rigid endoscope vs. flexible endoscope, used needs to be valued carefully. The probably most common complication during neuroendoscopic procedures with a rigid endoscope is fornix contusion. This can be avoided with the use of a flexible endoscope, which allows a safe navigation from the lateral to the fourth ventricle. For CC extending back to the roof of the third ventricle, a flexible endoscope might be preferred [24]. On the other hand, navigation within the ventricle using a flexible endoscope requires some experience, while the light intensity and optics are inferior and the working channels are more restricted when compared to a rigid endoscope [24]. A septum pellucidotomy must always be done with great caution, since if performed too cranially, the ipsilateral fornix might be damaged. In addition, due to impaired vision of the contralateral fornix, a septum pellucidotomy performed too anteriorly might damage the ipsilateral fornix. Rinsing of the ventricles in hydrocephalic patients and in neonates should be kept to a minimum, in order to avoid additional mechanical pressure to the surrounding brain and the ventricular structures (e.g., fornix and hypothalamus). The ideal trajectory is debated within the literature and should be adopted to the type of endoscopic procedure. Martinez-Moreno et al. have shown that the usage of neuronavigation leads to less displacement of important neuronal structures (fornix, hypo-/thalamus) when compared to manually planned trajectories [64]. Others suggested a supraorbital approach to the third ventricle for endoscopic resection of CC to avoid dissection of important neuronal structures and to provide better vision of the roof of the third ventricle. However, they recommend tailoring the approach according to the location of the CC (foraminal, foraminal/retroforaminal, and retroforaminal) [21].

### 4.5. Future Focus of Research for Neurocognition after Ventricular Neuroendoscopy

Focus of future research in terms of ventricular neuroendoscopy should include intraoperative damage to important structures (e.g., fornix), as well as neurocognitive complications and outcome. Studies analyzing neurocognition, by a trained neuropsychologist, before and after ventricular neuroendoscopy are essential, and such testing should be done routinely for all patients undergoing ventricular neuroendoscopic surgery. In addition, the patients' subjective opinion on their neurocognition, their quality of life, and their satisfaction of the completed surgery should be analyzed routinely, in the framework of studies, as well. The association of postoperative MRI, and specifically DTI, changes with neurocognition impairment is an additional aspect which is worthwhile investigating [63]. The debate, whether early treatment of obstructive hydrocephalus, or of other lesions within the 3^rd^ ventricle, is beneficial when compared to late treatment, should be further explored. The rate of cognitive complications after neuroendoscopic treatment of ventricular lesions compared to open microsurgical treatment remains ambiguous and needs further exploration. Studies with larger cohorts with neurocognitive assessment looking at neurocognitive complications, outcome, and quality of life before and after surgery are warranted for these purposes. In addition, the difference between neurocognitive deficits due to the pathology itself (e.g., hydrocephalus and CC) or due to intraoperative injury of important neuronal structures leading to neurocognitive impairment should be evaluated as well. Development of novel technologies such as pressure sensors, wide angle cameras, allowing better overview of adjacent structures, and smart robot-assisted endoscopy could be means to reduce critical structure damages. Lastly, a neuropsychologist should aim for a standardized neurocognitive test battery for patients undergoing ventricular neuroendoscopy, allowing an objective comparison of the different study results.

## 5. Conclusion

To date, the literature assessing and reporting on neurocognitive complications after ventricular neuroendoscopy is sparse. Most studies analyzing complications after ventricular neuroendoscopy do not report on neurocognitive complications. Of those series reporting on neurocognitive complications and/or outcome, the majority do not assess patients' neurocognition in a systematic matter. While neurocognitive decline after ventricular neuroendoscopy is a risk, depending on the pathology, one can expect an improvement in cognitive function after treatment. Based on this review, transient cognitive impairment occurs in 2% of the patients, while permanent cognitive deficits occur in 1% of the patients. However, these rates might be underestimated. Neurosurgeons should initiate systematic neurocognitive assessment before and after surgery, through trained neuropsychologists, in all patients undergoing ventricular neuroendoscopy. Patients need to be consented about the potential neurocognitive complications, especially postoperative amnesia or psychiatric symptoms (psychosyndrome), before surgery.

## Figures and Tables

**Table 1:** Results of all series describing cognitive complications and outcome after ventricular neuroendoscopic procedures.

Author	Year	No. of patients	Population	Pathologies included	Endoscopic procedure	Standardized assessment for cognitive complication	Type of cognitive complication	Percentage of transient cognitive complications (% (*n*))	Percentage of permanent cognitive complication (% (*n*))	Standardized assessment for cognitive outcome	Follow-up time (years)	Type of endoscope
Abdou and Cohen [18]	1998	13	Adult	CC	Resection	No	MI	23.1 (3)	0	No	4	R
Aref et al. [19]	2017	131	Adult	Various	ETV ± biopsy	No	ND	0.8 (1)	ND	No	ND	R
Boogaarts et al. [20]	2010	85	Adult	CC	Resection	No	MI, PS	7.8 (7)	1.2 (1)	No	4.4	R
Birski et al. [10]	2016	27	Mixed	CC	Resection	Yes	MI	3.7 (1)	0	Yes^§^	3.6	R
Brunori et al. [21]	2018	22	Adult	CC	Resection	No	MI	9.1 (2)	4.5 (1)	No	ND	R
Burtscher et al. [12]	2002	6	Adult	LIAS	ETV	Yes	None	0	0	Yes	1.5	R
Calisto et al. [22]	2014	20	Mixed	HH	Disconnection	No	MI	10 (2)	0	No	1	R
Charalampaki et al. [23]	2005	13	Mixed	SSC	Fenestration	No	PS	0	8 (1)	No	ND	R
Constantini et al. [2]	2013	293	Mixed	Tumor	Biopsy ± ETV	No	MI	0.4 (1)	0	No	ND	U
El-Ghandour [24]	2009	10	Adult	CC	Resection	No	MI	10 (1)	0	No	2	R
Eshra [25]	2018	16	Adult	CC	Resection	No	MI	18.8 (3)	0	No	0.4	R
Ferrer et al. [26]	1997	4	Adult	Tumor	ETV and biopsy	No	MI	25 (1)	0	No	ND	F
Girgis et al. [5]	2015	330	Mixed	Various	Various	No	MI	0	0.3 (1)	No	12.9	U
Hader et al. [27]	2014	13	Mixed	OHC	ETV	Yes	DEF	0	15.4 (2)	Yes	ND	U
Hayashi et al. [28]	2011	714	Mixed	Tumor	Biopsy	No	MI	0	0.4 (3^∗^)	No	1.9	B
Hellwig et al. [29]	2003	20	Mixed	CC	Resection	No	MI	ND	15 (3)	No	5.3	B
Hoffman et al. [30]	2013	58	Mixed	CC	Resection	No	MI	3.4 (2)	0	No	3.4	R
Hugelshofer et al. [3]	2015	31	ND	IVC	Fenestration	Yes	MI	3.2 (1)	0	Yes	2.4	R
Iacoangeli et al. [31]	2014	19	Adult	CC	Resection	No	MI	5.3 (1)	0	No	5.7	R
Ibanez-Botella et al. [32]	2014	24	Mixed	CC	Resection	No	MI	8.3 (2)	8.3 (2)	No	5.6	R
Isaacs et al. [33]	2016	163	Adult	Various HC	ETV	No	MI	0	0.6 (1)	No	8	B
Javadpour and Mallucci [34]	2004	11	Mixed	TG	ETV ± biopsy	No	CI	0	9 (1)	No	2.3	F
Krahenbuhl et al. [17]	2016	44	Mixed	Tumor	Biopsy ± ETV	No	Confusion	2.3 (1)^“^	0	No	4.1	R
Lacy et al. [14]	2009	10	Adult	OHC	ETV	Yes	None	0	0	Yes	2	U
Levine et al. [35]	2007	35	Mixed	CC	Resection	No	MI	11.4 (4)	0	No	7.8	F
Margetis et al. [36]	2014	77	Mixed	CC	Resection	No	MI	1.3 (1)	1.3 (1)	No	2.7	R
Mohanty et al. [37]	2011	87	Mixed	Tumor	ETV + biopsy	No	MI	0	0^∞^	No	1.9	R
Oertel et al. [38]	2009	134	Peds	OHC	Various	No	PS^×^	0	0.8 (1)	No	1	R
Oertel et al. [39]	2017	130	Mixed	Various	Combined procedures*^α^*	No	PS^″^	2.3 (3)	0	No	1.3	B
Parikh et al. [40]	2009	34	Mixed	Various	ETV + reservoir	No	MI, PS	0	5.9 (2)	No	2.2	U
Pinto et al. [41]	2009	11	Adult	CC	Nd:YAG laser resection	No	CI	0	0	Yes (ND)	2.75	R
Rodziewicz et al. [42]	2000	12	Mixed	CC	Resection	No	MI	8.3 (1)	0	No	3.6	R
Roth et al. [15]	2019	18*^μ^*	Adult	CC	Resection	Yes	MI	ND	0	Yes	2.9	U
Sribnick et al. [16]	2013	56	Mixed	CC	Resection	No	MI	10.7 (6)	10.7 (6)	No	1.2	R
Tirakotai et al. [43]	2004	22	Adult	CC	Resection	No	MI, PS^~^	4.5 (1)	4.5 (1)	No	ND	B
Torres-Corzo et al. [44]	2014	33	Mixed	FVOO	Magendie/Luschka foraminoplasty	No	MI*^β^*	0	0	No	2.3	F
Vorbau et al. [4]	2019	20	Mixed	CC	Resection	Yes	PS, MI	15 (3)	0	Yes	15.7	R
Wait et al. [45]	2013	16	Mixed	CC	Resection	No	MI	25 (4)	0	No	2.1	R
Yadav et al. [46]	2014	24	Mixed	CC	Resection	No	MI	0	4.2 (1)	No	3.1	R
Zohdi and El Kheshin [47]	2006	18	Mixed	CC	Resection	No	MI	16.7 (3)	0	No	4.2	R

No. = number; Peds = pediatric; CC = colloid cyst; LIAS = late onset idiopathic aqueduct stenosis; HH = hypothalamic hamartoma; SSC = suprasellar cyst; IVC = intraventricular cyst; OHC = obstructive hydrocephalus; HC = hydrocephalus; TG = tectal glioma; ETV = endoscopic third ventriculostomy; MI = memory impairment; CI = cognitive impairment; DEF = declined executive function; PS = psychosyndrome; ND = not defined; R = rigid; F = flexible; B = both rigid and flexible; U = unknown. *^α^*Combined procedures including ETV, septostomy, biopsy, aqueductoplasty, cyst fenestration, cyst resection, catheter removal, foraminotomy, and stent placement. Included were all endoscopies with at least two of these procedures combined in one setting. ^∗^Intraoperative fornix injury in 2 patients. ^“^Intraoperative unilateral fornix lesion in 3 patients, however not causing clinical symptoms. ^§^Cognitive assessment only in 10 out of 27 patients. ^×^One fornix lesion without neurocognitive impairment. ^″^12 fornix lesions (9 small contusions, 3 loss of structure, 1 with bleeding). Four patients showed transient deficits (3 cognitive) due to fornix lesion. *^μ^*Mixed cohort of microsurgical (*n* = 4) and endoscopic (*n* = 18) operated patients as well as conservatively treated patients (*n* = 13); 3 patients treated by endoscopy had fornix injury. ^∞^Fornix lesion described in 7 patients (7 mild, 1 significant). ^~^PS was transient; MI was permanent. *^β^*6 fornix lesions.

**Table 2:** Rates of cognitive complications by type of ventricular endoscopic surgery.

Procedure (*n* of studies)	Transient (%)	Permanent (%)	Transient (*n*/*n* all)	Permanent (*n*/*n* all)
ETV (5)	0	2.21	0/226	5/226
CC resection (20)	7.96	2.65	45/565	16/603
ETV ± biopsy (6)	0.70	0.23	4/570	1/439
Biopsy alone (1)	0	0.42	0/714	3/714
Cyst fenestration (2)	2.27	2.27	1/44	1/44
Foraminoplasty (2)	0	0	0/33	0/33
Hypothalamic hamartoma disconnection (1)	10	0	2/20	0/20
Combined procedures (1)	2.30	0	3/130	0/130
Various procedures (2)	0	0.43	0/464	2/464

ETV: endoscopic third ventriculostomy; CC: colloid cyst; *n*: number.

**Table 3:** Recommended neuropsychological test battery for neurocognitive evaluation before and after neuroendoscopic procedures.

Test	Function tested
Montreal Cognitive Assessment (MOCA) test	Memory recall, visuospatial abilities, executive functions, attention, concentration, working memory, language, orientation, and time
Clock-drawing test	Cognition
Language screening	Language ability
Boston Naming Test	Confrontational word retrieval, speech
Visual and verbal length of memory and working memory	Memory
Rey-Osterrieth Complex Figure (ROCF) test	Visuospatial abilities, memory, attention, planning, working memory, and executive functions
Verbal Learning and Memory (VLMT) test	Memory
Verbal and figural fluency	Nonverbal capacity for fluid and divergent thinking, ability to shift cognitive set, planning strategies, and executive ability
Stroop test	Object naming, executive functions, and concentration
Trail Making Test (TMT A & B)	Visual attention and task switching
Modified Wisconsin Card Sorting Test (mWCST)	Flexibility in the face of changing schedules of reinforcement
Test of Attentional Performance (TAP)	Attention, alertness, and split attention
